# Trends in osteoporosis and mean bone density among type 2 diabetes patients in the US from 2005 to 2014

**DOI:** 10.1038/s41598-021-83263-4

**Published:** 2021-02-12

**Authors:** Yingke Xu, Qing Wu

**Affiliations:** 1 Department of Epidemiology and Biostatistics, School of Public Health, University of Nevada, Las Vegas, Las Vegas, NV 89154 USA; 2 Nevada Institute of Personalized Medicine, College of Science, University of Nevada, Las Vegas, Las Vegas, NV 89154 USA

**Keywords:** Diabetes, Metabolic bone disease, Epidemiology

## Abstract

This study aimed to examine how bone health changed among T2DM patients in the past decade. Continuous National Health and Nutrition Examination Survey (NHANES) data from 2005–2006 to 2013–2014 were analyzed to examine the trends of bone mineral density (BMD) and the prevalence trends of osteoporosis osteopenia among T2DM patients and non-diabetic people aged 40 years and older. The age- and BMI-adjusted mean BMD of the femur neck for the four NHANES cycles decreased linearly in both T2DM patients and non-diabetic people (both P_linear trend_ ≤ 0.009). Among women with T2DM, the mean BMD in 2013–2014 was significantly lower than that in 2005–2006, even after adjusting for multiple covariates. During 2005–2014, the prevalence of osteoporosis among T2DM patients and non-diabetic people increased but with no significant linear trend (both P_linear trend_ > 0.05), while the prevalence of osteopenia in the two populations increased linearly (both P_linear trend_ < 0.04). Age- and BMI-adjusted mean BMD decreased in 2013–2014 in patients with T2DM and non-diabetic people, while the prevalence of osteoporosis and osteopenia increased in both groups.

## Introduction

Osteoporosis and low bone mass (osteopenia) affect approximately 200 million people across the world^1^, including 54 million people in the United States^2^. People with these conditions are prone to have fractures. Indeed, around 158 million people were at a high fracture risk in 2010, with that number predicted to double by 2014^3^. These related fractures will ultimately lead to many issues in those afflicted, including movement restriction, disability, and severe morbidity.

Diabetes is characterized by hyperglycemia resulting from defects in insulin secretion, insulin action, or both^4,5^. The number of people with diabetes worldwide has risen from 108 million in 1980 to 422 million in 2014^6^. Even more notably, type 2 diabetes mellitus (T2DM) accounts for 90–95% of all diabetes^7^. The prevalence of diabetes increased rapidly from 4.4 to 10.0% in the US between 1996 and 2015^8^, and more than 30 million suffer from T2DM^9^.

Osteoporosis and T2DM are affected by aging and often coexist in the elderly^10^. T2DM affects bone metabolism and strength by influencing osteoblast and osteoclast^11^. The imbalance between osteoblast and osteoclast might cause osteoporosis^11^. As well, T2DM might affect bone quality and quantity, leading to a change in the structural properties of bone mass^12^. T2DM affects bone homeostasis, so related fractures are considered a result of T2DM^12,13^. Several studies have already reported an increased fracture risk among T2DM patients^14–17^. Therefore, evaluating the bone health of individuals with T2DM is essential in preventing osteoporosis and related fractures. However, to our knowledge, how the bone health of T2DM patients changed in recent years remains unclear. As BMD is the most important single predictor for osteoporotic fractures, the dual-energy X-ray absorptiometry (DXA)-based BMD has been the benchmark technique used in osteoporosis diagnosis^18,19^. DXA-based BMD plays a crucial role in osteoporosis/osteopenia management and fracture risk assessment. Therefore, the present study aimed to test whether people with T2DM have had an elevated or decreased BMD in the past decades and compare the results to non-diabetic people. We also examined the trends of osteoporosis and osteopenia prevalence in T2DM patients and non-diabetic people.

## Methods

### Data source

Data were obtained from the continuous National Health and Nutrition Examination Survey (NHANES). The survey uses a complex, multistage probability design to select a nationally representative sample of non-institutionalized civilians in the US population^20^. NHANES collects data through interviews and physical examination. The interview includes demographic, socioeconomic, dietary, and health-related questions, while the examination consists of medical, dental, and physiological measurements. From 2005–2006, the survey started measuring femur and spine BMD; however, the BMD was not measured for NHANES in 2011–2012. Therefore, only four cycles (2005–2006, 2007–2008, 2009–2010, and 2013–2014) were included for this study. Subjects younger than 40 years old were excluded since participants are more likely to develop T2DM if they are 40 or over^21^.

### Human participants

NHANES study protocol was approved by the National Center for Health Statistics Research Ethics Review Board. Written informed consent was obtained for all adult participants. This secondary analysis was approved by the institutional review board at the University of Nevada, Las Vegas (#1004670). All research reported in this manuscript was performed in accordance with relevant guidelines/regulations.

### Diabetes mellitus

Subjects who had a positive response to the question, “Have you ever been told by a doctor that you have diabetes?” were defined as having a diagnosed diabetes. Individuals who reported taking pills to lower blood sugar were classified as having a diagnosed T2DM as well. This classification method was used^22^, and other study using NHANES data adopted this method as well^23^. Among participants without diagnosed diabetes, individuals who had a hemoglobin A1c level of 6.5% or higher, or a fasting plasma glucose level of 126 mg/dL or higher, or a 2-h plasma glucose level of 200 mg/dL or higher^5^, were all classified as having “undiagnosed diabetes.” Because we could not distinguish between type 1 and type 2 diabetes based on these lab results, all of the undiagnosed diabetes in this study was assumed to be T2DM since T2DM comprises the majority (90–95%) of diabetes^7^. Diagnosed T2DM and undiagnosed T2DM were combined as the T2DM group in this study. People were classified as non-diabetic if they did not have diagnosed or undiagnosed diabetes.

### BMD measurement

Femur neck BMD and spine BMD were measured using a Hologic QDR-4500A fan-beam densitometer during 2005–2010. Both BMDs were obtained with a Hologic Discovery model A densitometer (Hologic, Inc., Bedford, MA, USA) in 2013–2014. During 2005–2010, Hologic Discovery v12.4 and APEX v3.0 were used for analyzing the femur and spine scans, respectively. APEX v4.0 was used for the analysis in the two regions in 2013–2014. In this study, we focused on analyzing the femur neck BMD primarily because BMD at the femur neck has the highest predictive value for hip fracture, and the hip is the site of highest clinical relevance^24^. We used femur neck BMD over spine BMD in this study because of significant differences in BMD measures between using Discovery v12.4 during 2005–2010 and using APEX v4.0 in 2013–2014, with the exception of the femur neck^25^. In NHANES, DXA scans were used for the BMD measurement since the system has a number of advantages, the primary being a consensus that BMD results can be interpreted using the World Health Organization T-score definition of osteoporosis, thus having a proven ability to predict fracture risk^26^.

### Definition for osteoporosis and osteopenia

In this study, the diagnosis of osteoporosis or osteopenia was based on T-score results. T-scores were calculated as (BMD_measured_ − mean BMD_reference_)/SD_reference_. Osteoporosis was defined as a T-score of BMD ≤  − 2.5, and osteopenia was defined as − 2.5 < T-score ≤ − 1^27^. Consistent with the International Society for Clinical Densitometry’s corresponding guidelines, the reference group for calculating these scores for the femur neck consisted of 20–29 years old non-Hispanic Caucasian women from NHANES III^28^. In our study, subjects who lacked valid BMD data were excluded.

### Other variables

Age, sex, race/ethnicity, smoking status, physical activities, fracture history, and family history were ascertained by questionnaire. For the race/ethnicity groups, “Mexican American” and “Other Hispanic” were merged into a single group called “Hispanic,” and the remaining groups were “non-Hispanic White,” “non-Hispanic Black,” and “non-Hispanic other,” respectively. BMD-related variables, including body mass index (BMI), previous fracture^29,30^, smoking status^31,32^, physical activity^33,34^, and family history of osteoporosis^35^, were considered for analysis. BMI was derived from measured weight in kilograms, divided by the square of height in meters. Individuals who had suffered a broken or fractured hip, wrist, or spine were considered as having a previous fracture. Smoking status was categorized into smokers and non-smokers. Smokers were respondents who had smoked at least 100 cigarettes during their lifetime; otherwise, subjects were defined as non-smokers. Self-reported physical activity was categorized as “inactive” and “active.” Participants who were sedentary or only performed basic activities, which refers to light-intensity activities like standing and walking slowly, were considered to be inactive; otherwise, the individuals were classified as active^36^. In the present study, the participants were defined as having a family history of osteoporosis if their parent(s) ever had the disease.

### Statistical analysis

Sampling weights were utilized to account for unequal selection probabilities, nonresponse, and non-coverage^37^. The age- and BMI-adjusted mean BMD at the femur neck, prevalence of osteoporosis and osteopenia, and corresponding 95% confidence interval in every survey cycle for both T2DM patients and non-diabetic people were calculated based on weighted data. Standard errors, which were employed to calculate 95% confidence intervals, were estimated using Taylor series linearization. The US 2000 Census was used as the standard population for age adjustment. Tests for linear trend over the four survey cycles were conducted using orthogonal contrast. Multiple linear regression was used to examine the BMD trend while holding other variables constant. The standardized coefficient from linear regression was employed to examine each variable’s relative importance in the regression model for BMD prediction. The survey cycle was included as a categorical variable in the multiple linear regression in order to determine if the mean BMD in 2013–2014 differed from previous survey cycles after adjusting for major confounders. We also conducted separate sensitivity analyses to examine the mean BMD trends across the four survey cycles in T2DM patients and non-diabetic population after adjusting for age and weight (instead of BMI) and in diagnosed and undiagnosed T2DM patients after adjusting for age and BMI. All analyses were conducted using SAS version 9.4 (SAS Institute, Cary, NC, USA).

## Results

The number of eligible participants in this study was 11,901, and their characteristics by survey cycles are presented in Table 1. During the four survey cycles, the mean age was around 62 years for T2MD patients and 56 years for the non-diabetic population. From 2005 to 2014 mean BMI increased among T2DM patients (from 29.93 to 31.38 kg/m^2^), as well as among the non-diabetic population (from 27.75 to 28.27 kg/m^2^). In each survey cycle, more than half of T2DM patients were men, while most non-diabetic participants were women. Over the four survey cycles, the percentage of physical inactivity increased from 14.04 to 34.61% in T2DM patients and from 9.89 to 23.80% in the non-diabetic population.

**Table 1:** Weighted characteristics of subjects aged 40 and older, among T2DM patients and non-diabetic population in 4 NHANES (2005–2006, 2007–2008, 2009–2010, and 2013–2014).

Variable	Survey cycle							
	2005–2006		2007–2008		2009–2010		2013–2014	
	With T2DM (N = 409)	Non-diabetic (N = 1853)	With T2DM (N = 720)	Non-diabetic (N = 2465)	With T2DM (N = 734)	Non-diabetic (N = 2619)	With T2DM (N = 674)	Non-diabetic (N = 2427)
Age, mean (SD), years	62.12 ± 0.85	55.51 ± 0.71	61.47 ± 0.51	55.60 ± 0.32	62.47 ± 0.72	55.84 ± 0.39	61.22 ± 0.71	56.42 ± 0.34
BMI, mean (SD), kg/m^2^	29.93 ± 0.30	27.75 ± 0.21	31.32 ± 0.29	27.74 ± 0.12	31.23 ± 0.27	27.86 ± 0.14	31.38 ± 0.26	28.27 ± 0.19
**Gender, N (%)**								
Men	227 (53.26)	975(48.53)	394 (51.87)	1209 (46.39)	377 (52.12)	1323 (48.08)	371 (57.04)	1162 (47.81)
Women	182 (46.74)	878 (51.47)	326 (48.13)	1256 (53.61)	357 (47.88)	1296 (51.92)	303 (42.96)	1265 (52.19)
**Race, N (%)**								
Hispanic	115 (13.33)	323 (7.05)	208 (11.92)	617 (9.21)	260 (15.67)	678 (9.76)	192 (16.44)	496 (10.26)
NH White	166 (66.58)	1108 (80.19)	316 (67.67)	1315 (76.36)	293 (64.45)	1411 (76.17)	234 (61.41)	1132 (73.44)
NH Black	112 (14.60)	356 (8.13)	174 (14.47)	437 (8.75)	134 (12.04)	418 (8.77)	156 (13.26)	462 (9.38)
NH other	16 (5.49)	66 (4.63)	22 (5.94)	96 (5.68)	47 (7.84)	112 (5.29)	92 (8.88)	337 (6.92)
Previous fracture, N (%)	55 (15.79)	272 (14.97)	89 (13.76)	328 (14.49)	74 (11.22)	306 (12.02)	73(12.80)	260 (12.34)
Smoking, N (%)	226(52.20)	995 (53.61)	380 (52.21)	1245 (48.77)	362 (49.04)	1287 (46.54)	326 (50.96)	1111(45.30)
Physical inactivity, N (%)	73 (14.04)	244 (9.89)	310 (41.22)	708 (22.40)	308 (38.44)	733 (22.57)	226 (34.61)	612 (23.80)
Family history of osteoporosis, N (%)	28 (10.17)	213 (14.29)	67 (12.71)	334 (16.74)	61 (8.97)	292 (14.47)	74 (13.51)	312 (16.84)

### Adjusted mean femur neck BMD

**Figure 1:**
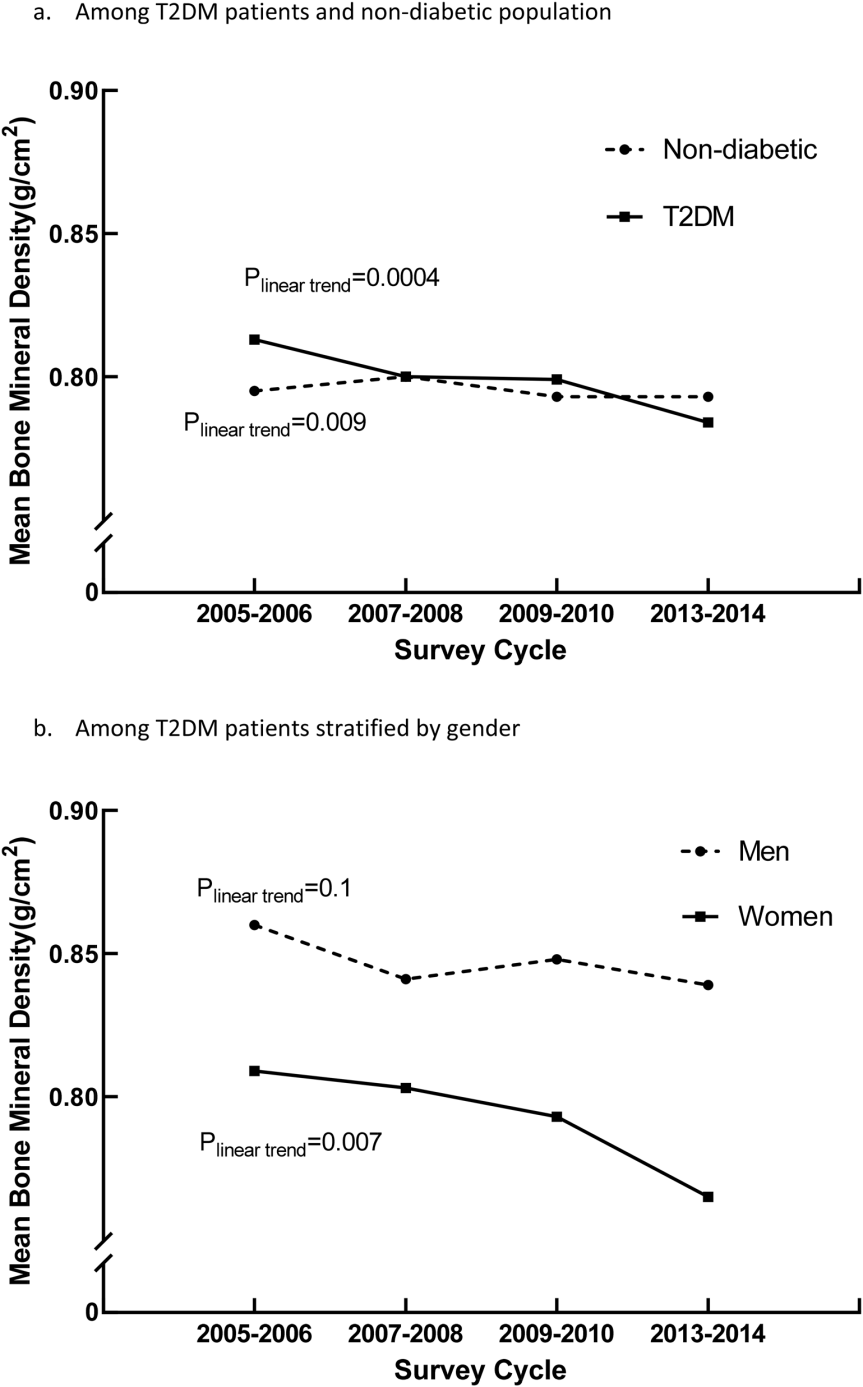
Age and body mass index-adjusted mean bone mineral density in 4 NHANES (2005–2006, 2007–2008, 2009–2010, and 2013–2014).

The age- and BMI-adjusted mean BMD of T2DM patients and non-diabetic people in the four survey cycles are shown in Fig. 1a. For T2DM patients, the mean BMD decreased linearly from 0.813 g/cm^2^ (95% CI 0.796–0.829 g/cm^2^) to 0.784 g/cm^2^ (95% CI 0.771–0.796 g/cm^2^) during 2005–2014 (P_linear trend_ = 0.004). Meanwhile, the mean BMD of non-diabetic also decreased linearly during the four survey cycles (P_linear trend_ = 0.0009), from 0.795 to 0.773 g/cm^2^.

The results of age- and BMI- adjusted mean BMD of T2DM patients stratified by gender are presented in Fig. 1b. Among patients with T2DM, women had a lower mean BMD than men during the four survey cycles. In 2005–2014, the mean BMD of women with T2DM linearly decreased (P_linear trend_ = 0.007), but no significant linear trend (P_linear trend_ = 0.1) was observed among men with T2DM.

The age- and BMI-adjusted mean BMD of the non-diabetic population stratified by gender are presented in Supplementary Figure 1. Significant linear trends were observed for both genders during 2005–2014 (both P_linear trend_ ≤ 0.005). Our sensitivity analysis revealed that the results of age- and weight-adjusted mean BMD (Supplementary Figure 2) were similar to age- and BMI-adjusted mean BMD. In addition, the age- and BMI-adjusted mean BMD (Supplementary Figure 3) decreased linearly in people with diagnosed T2DM (P _linear trend_ = 0.02), but not for people with undiagnosed T2DM (P_linear trend_ = 0.39) during 2005–2014.

### Prevalence of osteoporosis and osteopenia

The age- and BMI-adjusted prevalence of osteoporosis and osteopenia of T2DM patients and non-diabetic people from 2005 to 2014 are presented in Table 2. The adjusted prevalence of osteoporosis among T2DM patients increased from 3.13% (95% CI 1.39–4.87%) to 6.10% (95% CI 4.47–7.75%) during 2005–2014, and the linear trend was close to being statistically significant (P_linear trend_ = 0.054). For non-diabetic individuals, the adjusted prevalence of osteoporosis was stable during this period (P_linear trend_ = 0.35). The adjusted osteopenia prevalence of T2DM patients and non-diabetic people had a significant increase in the linear trend (both P_linear trend_ ≤ 0.04).

**Table 2:** Age- and BMI-adjusted prevalence of osteoporosis and osteopenia among T2DM patients and non-diabetic population in 4 NHANES (2005–2006, 2007–2008, 2009–2010, and 2013–2014).

Prevalence	Survey cycle				
	2005–2006	2007–2008	2009–2010	2013–2014	P value for linear trend
**Osteoporosis**					
T2DM	3.13 (1.39–4.87)	4.50 (1.55–7.44)	3.10 (1.75–4.44)	6.10 (4.47–7.75)	0.054
Non diabetic	4.03 (3.26–4.79)	3.03 (2.30–3.74)	3.81 (3.18–4.46)	4.38 (3.39–5.37)	0.35
**Osteopenia**					
T2DM	27.83 (21.62–34.03)	31.14 (26.75–35.54)	32.56 (27.15–37.97)	35.49 (30.06–40.92)	0.04
Non diabetic	32.23 (29.81–34.64)	30.27 (28.14–32.41)	32.82 (30.81–34.84)	38.20 (35.51–40.90)	0.0005

The age- and BMI-adjusted prevalence of osteoporosis and osteopenia among T2DM patients by gender are presented in Table 3. The linear trend of the osteoporosis prevalence among women approached the borderline of significance (P_linear trend_ = 0.08) but was non-significant for men (P_linear trend_ = 0.14). The osteoporosis prevalence among women with T2DM increased from 3.95% (95% CI 0–7.98%) to 10.13% (95% CI 6.06–14.21%) during the four survey cycles. For the prevalence of osteopenia among T2DM patients, a significant increase in linearly trend was observed in women (P_linear trend_ = 0.04), but not in men (P_linear trend_ = 0.35). The prevalence of osteoporosis and osteopenia in non-diabetic people by gender is presented in Supplementary Table 1. The prevalence of osteoporosis in non-diabetic men and women was stable during the four survey cycles (both P_linear trend_ > 0.29). However, significant linear trends in osteopenia prevalence were observed in non-diabetic men and women during the four survey cycles (both P_linear trend_  ≤  0.004).

**Table 3:** Age- and BMI-adjusted prevalence of osteoporosis and osteopenia among T2DM patients stratified by gender in 4 NHANES (2005–2006, 2007–2008, 2009–2010, and 2013–2014).

Prevalence	Survey cycle				
	2005–2006	2007–2008	2009–2010	2013–2014	P value for linear trend
**Osteoporosis**					
Men	0.02 (0–0.30)	2.29 (0–4.87)	1.31 (0–2.63)	1.21 (0.42–2.00)	0.14
Women	3.95 (0–7.98)	4.95 (1.67–8.23)	3.19 (0.81–5.57)	10.13 (6.06–14.21)	0.08
**Osteopenia**					
Men	21.34 (14.34–28.34)	22.44 (18.05–26.82)	20.98 (14.68–27.28)	27.14 (19.01–35.26)	0.35
Women	25.22 (18.82–31.62)	31.13 (25.08–37.17)	35.34 (27.52–43.15)	35.42 (26.82–44.02)	0.04

### Multiple linear regression

For women with T2DM, older age and previous fractures were significantly associated with decreased BMD, based on the multiple linear regression (both p ≤ 0.03; Supplementary Table 2). For men with T2DM, older age, family history of osteoporosis, physical inactivity, and smoking were associated with significant BMD reduction (all p < 0.007; Supplementary Table 3). The standardized coefficient revealed that age played a more critical role than other risk factors in lowering BMD for T2DM patients of both sexes. The selected covariates explained around 35% variance of BMD at the femur neck in women and about 27% in men. The mean BMD of women with T2DM in 2013–2014 was significantly lower than in 2005–2006 (p = 0.0007; Supplementary Table 2), even after adjusting for various confounders. The results of non-diabetic individuals, both women and men, are respectively presented in Supplementary Table 4 and Table 5. For non-diabetic people, a family history of osteoporosis, age, and physical inactivity had a significant negative impact on BMD in both men and women.

## Discussion

In this study, decreasing BMD trends at the femur neck were observed among T2DM patients and non-diabetic people during 2005–2014. In addition, the prevalence of osteoporosis and osteopenia among subjects with T2DM increased correspondingly in the four survey cycles. Specifically, a significant increasing linear trend in the prevalence of osteoporosis and osteopenia was observed among women with T2DM. These consistent results demonstrate that an unfavorable trend of change in bone health in T2DM patients may be occurring in the United States.

Compared to our prior study regarding the US BMD trend in 2005–2014^38^, the present research further examined the BMD trend, osteoporosis, and osteopenia in the US's T2DM population. There is a paucity of research investigating the BMD trend and the pattern of osteoporosis and osteopenia in T2DM patients, specifically a lack of data comparing the trend between individuals with T2DM and those without diabetes. In this study, T2DM patients had a higher BMD than non-diabetic people, consistent with Dr. Oei et al.’s research^16,39^. Among T2DM patients, men had a higher BMD mean than women. The possible reason for this is that men have a larger bone mass than women^40^. Notably, the mean BMD in 2013–2014 among women with T2DM was significantly lower than the mean BMD in 2005–2006. Such a difference remained when adjusted for multiple related factors, implying proper intervention might be needed. Although such a linear trend of BMD was not observed among men in the current study, attention should be paid to men since a previous study showed that men with diabetes had a higher risk of fracture^41^. Our findings of non-diabetic people were consistent with a recent study conducted by Dr. Chi Chen et al. that found a declining BMD trend among adults with normal glucose regulation^42^. The decreasing mean BMD trend of this study demonstrates that the bone health of T2DM patients and non-diabetic people might have deteriorated significantly in recent years. In Dr. Black and colleagues’ study^43^, people with the lowest quartile of BMD almost had fivefold increased hip fracture risk during 25 years follow-up when compared with those with the highest quartile of BMD. Therefore, as shown in the current study, primary prevention and treatment should be conducted among the non-diabetic population with the lowest quartile BMD.

The multiple linear regression results indicated that sex, age, race, previous fracture, BMI, smoking, and physical activity are significant for T2DM patients^6,8,27,29–35,44–48^. Thus these related risk factors might partially contribute to the declining trend in mean BMD. The percentage of physical inactivity increased from 14.04 to 34.61% among T2DM and from 9.89 to 23.80% among non-diabetics. Given the association between physical inactivity and lower BMD^34^, the increased physically inactive lifestyle might also explain the decreasing BMD trends occurring during the four survey cycles. However, the decreased BMD in 2013–2014 among women with T2DM remained significant after controlling for major confounding effects in the multiple regression analysis. Thus other factors such as antidiabetic medication of T2DM patients may play a role in the observed trend^49,50^. For example, Dr. Monami and colleagues found an increased risk of fracture among T2DM patients who used insulin^51^. Patients with insulin usually have complications such as microvascular disease, which might impair the bone quality and individual balance^52^, thus increasing fracture risk. Additionally, Schwartz et al. reported an increased bone loss in diabetic women who had taken thiazolidinediones medication (TZD), including rosiglitazone, pioglitazone, and troglitazone^49^. Several studies investigated the effect of TZD on bone metabolism and found it is associated with increased adipogenesis and impaired osteoblastogenesis, which might lead to impaired bone formation and ultimately to fractures^53^. Other studies found that two medications, exenatide (a glucagon-like peptide 1 receptor agonist) and dapagliflozin (a sodium glucose cotransporter-2 inhibitor) increased the risk of bone fractures^54,55^, while dipeptidyl peptidase-4 inhibitors were associated with decreased fracture risk^26,54,56^. Further research is warranted to explain the overall declining BMD trend in T2DM patients.

The underlying pathogenic mechanism of bone fragility in T2DM is complex and not fully understood^12^. Not only decreased bone mass but also bone microstructure might contribute to bone fracture. Typically, increased cortical porosity and reduced cortical density lead to bone structure change^52^. Prior studies found that T2DM patients had greater cortical porosity^57,58^, but lower cortical bone density^59^. Therefore, bone resistance to mechanical stress among T2DM patients will be increased, which will result in an increased risk of fracture.

Several limitations should be acknowledged in interpreting the finding of this study. First, many of the NHANES participants were excluded from BMD measurement due to hip fractures, pregnancy, or other reasons. However, nonresponse in the examination data was accounted for by sampling weights in NHANES. Second, information about T2DM treatment, which might impact bone health, is limited in NHANES. For example, TZD is widely prescribed for the treatment of T2DM, and accumulating evidence indicates that TZD could cause bone loss and increase fracture risk in humans, specifically in women^49^. However, too few participants reported this information, so we cannot analyze it to yield valid results. Third, the evaluation of the trend in hemoglobin A1c among T2DM patients would be informative since several studies reported the association between poor glycemic control and increased risk of fractures^60–63^. However, the missing value of hemoglobin A1c from NHANES is too much (around 80%) to get an accurate evaluation. Finally, the study design of NHANES is cross-sectional, which restricts the assessment of causal relationships.

Osteoporosis and low BMD lead to fractures, which cause severe consequences for both individual patients and health care systems^24^. Osteoporosis-related fractures often lead to decreased quality of life, disability, and even death for patients and are also associated with $20 billion in expenses in the US^64^. Due to T2DM patients having normal or higher BMD, BMD-based T-score might underestimate the fracture risk of T2DM patients, and evaluation of bone health and osteoporosis diagnosis among them might be challenging^52^. Derived from continuous NHANES data, our findings regarding osteoporosis trends and mean BMD among T2DM patients can be used to inform public health policy and thus contribute to needed reform. Policy intervention may help to reduce risk factors associated with the downward trend of bone health inT2DM patients.

## Conclusion

In summary, a decreasing age- and BMI-adjusted mean BMD trend has been observed in T2DM patients and the non-diabetic population in recent years. The unfavorable trend indicates a future downward shift in the bone health of T2DM patients, so bone health should be monitored in diabetic patients. Therefore, additional studies are warranted to understand the decreasing BMD trend among T2DM patients more thoroughly to prevent fractures and their subsequent deleterious consequences on individuals with diabetes.

## Supplementary Information

Supplementary Information.
