# Supplementary material for: Trends in osteoporosis and mean bone density among type 2 diabetes patients in the US from 2005 to 2014
Source: Sci Rep. 2021 Feb 12;11:3693. doi: 10.1038/s41598-021-83263-4 (PMC7881186; doi:10.1038/s41598-021-83263-4)
Supplement: Supplementary file 1 — Supplementary Information. [file 41598_2021_83263_MOESM1_ESM.docx]

**Supplementary Material**

**Trends in osteoporosis and mean bone density among in the US type 2 diabetes patients from 2005 to 2014**

Yingke Xu, MSPH.

Qing Wu, M.D., Sc.D.

Author Affiliations: Department of Epidemiology and Biostatistics, School of Public Health (Y.X., Q.W.), Nevada Institute of Personalized Medicine (Y.X., Q.W.), College of Science, University of Nevada, Las Vegas, Las Vegas, Nevada.

Corresponding author: Qing Wu, MD, Sc.D., Nevada Institute of Personalized Medicine, College of Science, Department of Epidemiology and Biostatistics, School of Public Health, University of Nevada, Las Vegas, Las Vegas, NV 89154 (qing.wu@unlv.edu, Phone: 702-895-1439, Fax: 702- 895-3094, ORCID ID: 0000-0003-4679-8903)

**Supplementary Figure 1** Age and body mass index-adjusted mean bone mineral density among nondiabetic population by gender in 4 NHANES (2005–2006, 2007–2008, 2009–2010, and 2013–2014)

**Supplementary Figure 2** Age and weight-adjusted mean bone mineral density among T2DM patients and nondiabetic population in 4 NHANES (2005–2006, 2007–2008, 2009–2010, 2013–2014)

**Supplementary Figure 3** Age and body mass index-adjusted mean bone mineral density among T2DM patients by diagnostic status in 4 NHANES (2005–2006, 2007–2008, 2009–2010, and 2013–2014)

**Supplementary Table 1** age- and BMI-adjusted prevalence of osteoporosis and osteopenia among nondiabetic population stratified by gender in 4 NHANES (2005–2006, 2007–2008, 2009–2010, and 2013–2014)

|  | 2005-2006 | 2007-2008 | 2009-2010 | 2013-2014 | P value for linear trend |
| --- | --- | --- | --- | --- | --- |
| Osteoporosis |  |  |  |  |  |
| men | 1.73 (1.21-2.25) | 1.28 (0.65-1.91) | 1.36 (0.83-1.89) | 2.12 (1.55-2.69) | 0.29 |
| women | 6.40 (4.97-7.83) | 4.64 (3.58-5.70) | 6.22 (4.89-7.56) | 6.68 (4.70-8.68) | 0.52 |
| Osteopenia |  |  |  |  |  |
| men | 23.87 (21.93-25.80) | 23.63 (20.88-26.38) | 25.52 (22.93-28.10) | 29.62 (26.30-32.94) | 0.001 |
| women | 41.68 (38.49-44.87) | 38.21 (35.67-40.75) | 41.46 (38.92-44.00) | 47.81 (44.87-50.76) | 0.004 |

**Supplementary Table 2** Multiple linear regression analysis of mean bone mineral density among women with T2DM in 4 NHANES (2005–2006, 2007–2008, 2009–2010, and 2013–2014)

| **parameter** | | **Coefficient** | **Standardized coefficient** | ***p* value** |
| --- | --- | --- | --- | --- |
| Survey cycle^a^ | 2005–2006 | 0.0485 | 0.1189 | 0.0007 |
|  | 2007–2008 | 0.0414 | 0.1155 | 0.003 |
|  | 2009–2010 | 0.0284 | 0.0143 | 0.05 |
| Age | | -0.0050 | -0.3729 | <0.0001 |
| Race^a^ | Hispanic | -0.0087 | -0.0196 | 0.44 |
|  | Non-Hispanic black | 0.0654 | 0.1497 | <0.0001 |
|  | Non-Hispanic other | 0.0175 | 0.0309 | 0.33 |
| Previous fracture^a^ | | -0.0398 | -0.0840 | 0.03 |
| Family history of osteoporosis ^a^ | | -0.0010 | -0.0022 | 0.94 |
| Physical inactivity ^a^ | | -0.0001 | -0.0004 | 0.99 |
| smoking ^a^ | | -0.0099 | -0.0309 | 0.43 |
| BMI | | 0.0074 | 0.3045 | <0.0001 |

^a^Survey cycle of 2013–2014, non-Hispanic white, no previous fracture, no family history of osteoporosis, physically active, and no smoking were the reference groups for the corresponding categorical variables, R^2^ = 0.3498

**Supplementary Table 3** Multiple linear regression analysis of mean bone mineral density among men with T2DM in 4 NHANES (2005–2006, 2007–2008, 2009–2010, and 2013–2014)

| **parameter** | | **Coefficient** | **Standardized coefficient** | ***p* value** |
| --- | --- | --- | --- | --- |
| Survey cycle^a^ | 2005–2006 | 0.0189 | 0.0502 | 0.06 |
|  | 2007–2008 | 0.0060 | 0.0178 | 0.59 |
|  | 2009–2010 | 0.0109 | 0.0328 | 0.35 |
| Age | | -0.0023 | -0.1751 | <0.0001 |
| Race^a^ | Hispanic | 0.0033 | 0.0078 | 0.75 |
|  | Non-Hispanic black | 0.0895 | 0.1965 | <0.0001 |
|  | Non-Hispanic other | 0.0233 | 0.0381 | 0.20 |
| Previous fracture^a^ | | -0.0286 | -0.0667 | 0.05 |
| Family history of osteoporosis ^a^ | | -0.0426 | -0.0871 | 0.007 |
| Physical inactivity ^a^ | | -0.0225 | -0.0774 | 0.01 |
| smoking ^a^ | | -0.0207 | -0.0703 | 0.002 |
| BMI | | 0.0103 | 0.3821 | <0.0001 |

^a^Survey cycle of 2013–2014, non-Hispanic white, no previous fracture, no family history of osteoporosis, physically active, and no smoking were the reference groups for the corresponding categorical variables, R^2^ = 0.2650

**Supplementary Table 4** Multiple linear regression analysis of mean bone mineral density among nondiabetic women in 4 NHANES (2005–2006, 2007–2008, 2009–2010, and 2013–2014)

| **parameter** | | **Coefficient** | **Standardized coefficient** | ***p* value** |
| --- | --- | --- | --- | --- |
| Survey cycle^a^ | 2005–2006 | 0.0173 | 0.0539 | 0.0012 |
|  | 2007–2008 | 0.0243 | 0.0783 | 0.0002 |
|  | 2009–2010 | 0.0122 | 0.0393 | 0.03 |
| Age | | -0.0049 | -0.4292 | <0.0001 |
| Race^a^ | Hispanic | -0.0005 | -0.0011 | 0.92 |
|  | Non-Hispanic black | 0.0684 | 0.1444 | <0.0001 |
|  | Non-Hispanic other | -0.0219 | -0.0379 | 0.003 |
| Previous fracture^a^ | | -0.0330 | -0.0808 | <0.0001 |
| Family history of osteoporosis ^a^ | | -0.0110 | -0.0333 | 0.02 |
| Physical inactivity ^a^ | | -0.0113 | -0.0353 | 0.006 |
| smoking ^a^ | | -0.0059 | -0.0217 | 0.16 |
| BMI | | 0.0081 | 0.3613 | <0.0001 |

^a^Survey cycle of 2013–2014, non-Hispanic white, no previous fracture, no family history of osteoporosis, physically active, and no smoking were the reference groups for the corresponding categorical variables, R^2^ = 0.4029

**Supplementary Table 5** Multiple linear regression analysis of mean bone mineral density among nondiabetic men in 4 NHANES (2005–2006, 2007–2008, 2009–2010, and 2013–2014)

| **parameter** | | **Coefficient** | **Standardized coefficient** | ***p* value** |
| --- | --- | --- | --- | --- |
| Survey cycle^a^ | 2005–2006 | 0.0227 | 0.0730 | 0.0008 |
|  | 2007–2008 | 0.0310 | 0.1000 | <0.0001 |
|  | 2009–2010 | 0.0269 | 0.0882 | 0.001 |
| Age | | -0.0031 | -0.2573 | <0.0001 |
| Race^a^ | Hispanic | 0.0073 | 0.0164 | 0.16 |
|  | Non-Hispanic black | 0.0838 | 0.1778 | <0.0001 |
|  | Non-Hispanic other | -0.0144 | -0.0249 | 0.07 |
| Previous fracture^a^ | | -0.0205 | -0.0544 | 0.004 |
| Family history of osteoporosis ^a^ | | -0.0316 | -0.0714 | <0.0001 |
| Physical inactivity ^a^ | | -0.0244 | -0.0678 | <0.0001 |
| smoking ^a^ | | -0.0051 | -0.0192 | 0.27 |
| BMI | | 0.0090 | 0.3151 | <0.0001 |

^a^Survey cycle of 2013–2014, non-Hispanic white, no previous fracture, no family history of osteoporosis, physically active, and no smoking were the reference groups for the corresponding categorical variables, R^2^ = 0.2419
